# Association of Health Conditions and Health Service Utilization With Intimate Partner Violence Identified via Routine Screening Among Middle-Aged and Older Women

**DOI:** 10.1001/jamanetworkopen.2020.3138

**Published:** 2020-04-21

**Authors:** Lena K. Makaroun, Emily Brignone, Ann-Marie Rosland, Melissa E. Dichter

**Affiliations:** 1 VA Center for Health Equity Research and Promotion, Pittsburgh, Pennsylvania; 2 VA Pittsburgh Healthcare System, Pittsburgh, Pennsylvania; 3 Department of Medicine, University of Pittsburgh, Pittsburgh, Pennsylvania; 4 Corporal Michael J. Crescenz VA Medical Center, Philadelphia, Pennsylvania; 5 Temple University School of Social Work, Philadelphia, Pennsylvania; 6 VA Center for Health Equity Research and Promotion, Philadelphia, Pennsylvania

## Abstract

**Importance:**

The US Preventive Services Task Force recently determined that there is insufficient evidence to recommend routine screening for intimate partner violence (IPV) in women who are middle-aged and older. Certain Veterans Health Administration (VHA) clinics have been routinely screening women of all ages for IPV since 2014.

**Objectives:**

To examine the proportion of women older than childbearing age (ie, ≥45 years) who have positive results when routinely screened for past-year IPV at VHA clinics and to evaluate the associations of a positive screening result with health conditions and health service utilization.

**Design, Setting, and Participants:**

This cohort study included 4481 women aged 45 years and older who were screened for past-year IPV in 13 VHA outpatient clinics in 11 states between April 2014 and April 2016. Data analysis was conducted from March 2019 to August 2019.

**Exposure:**

Positive screening result for past-year IPV.

**Main Outcomes and Measures:**

Mental and physical health conditions (identified using *International Classification of Diseases, Ninth Edition *[*ICD*-*9*] and *ICD*-*10* codes from VHA medical record data) and VHA health services utilization (identified using inpatient and outpatient VHA encounter data) in the 20 months after screening.

**Results:**

In this study, 2937 of 4481 women (65.5%) were middle-aged (ie, aged 45 to 59 years), and 1544 (34.5%) were older (ie, aged ≥60 years), with 1955 (43.6%) black participants. A total of 255 middle-aged women (8.7%; mean [SD] age, 51 [4] years) and 79 older women (5.1%; mean [SD] age, 64 [5] years) screened positive for past-year IPV. In adjusted logistic regression models among middle-aged women, screening positive for IPV was associated with subsequent diagnoses of anxiety (adjusted odds ratio [aOR], 2.00; 95% CI, 1.50-2.70; *P* < .001), depression (aOR, 2.30; 95% CI, 1.80-3.00; *P* < .001), posttraumatic stress disorder (aOR, 2.30; 95% CI, 1.80-3.00; *P* < .001), suicidal ideation and/or behavior (aOR, 3.80; 95% CI, 2.10-6.90; *P* < .001), and substance use disorder (aOR, 2.50; 95% CI, 1.80-3.50; *P* < .001). Similar but attenuated associations were seen for older women (eg, substance use disorder: aOR, 2.20; 95% CI, 1.10-4.40; *P* = .04). In adjusted negative binomial regression models among middle-aged women, screening positive for IPV was associated with a higher rate of subsequent psychosocial (eg, mental health) visits (adjusted rate ratio [aRR], 2.40; 95% CI, 2.00-2.90; *P* < .001), primary care visits (aRR, 1.20; 95% CI, 1.10-1.30; *P* < .001), and emergency department visits (aRR, 1.50; 95% CI 1.20-1.80; *P* < .001). Older women screening positive for IPV had a higher rate of psychosocial visits (aRR, 1.90; 95% CI, 1.30-2.70; *P* < .001) but not of other visit types.

**Conclusions and Relevance:**

To our knowledge, this study was the largest to evaluate routine screening for IPV among women aged 45 years and older, and it found that IPV remained prevalent and was associated with morbidity for these women. Screening for IPV in women older than 44 years may improve detection and provision of evidence-based services to this growing population.

## Key Points

**Question:**

How often do middle-aged and older women have positive results when undergoing routine screening for intimate partner violence (IPV), and what health-related outcomes are associated with positive results in these age groups?

**Findings:**

In this cohort study of 4481 female veterans aged 45 years and older who were screened for past-year IPV, 8.7% of those aged 45 to 59 years and 5.1% of those aged 60 years and older screened positive. Having screened positive for IPV was associated with mental and physical health outcomes as well as increased health service utilization over the subsequent 20 months.

**Meaning:**

Performing routine screening for IPV among women in middle age and older may improve detection and service delivery in this underserved population.

## Teaser

This cohort study examines the proportion of women aged 45 years and older who screen positive when routinely screened for past-year intimate partner violence at Veterans Health Administration clinics and evaluates the associations of positive screening results with health conditions and health service utilization.

## Introduction

Intimate partner violence (IPV), defined as psychological, physical, or sexual aggression by a current or former intimate partner, is experienced by an estimated 5.3 million women in the US annually.^1^ Among women of childbearing age, experiencing IPV has been found to be associated with negative mental and physical health outcomes, including depression, posttraumatic stress disorder (PTSD), chronic pain, gastrointestinal disorders, heart disease, injury, substance use disorders, and sexually transmitted infections.^1,2,3^ While younger women who have experienced IPV may be less likely to use specific types of health services, such as maternal care,^4^ those with lifetime exposure to IPV have overall higher health service utilization and health care costs.^5,6^ For older women, who are more likely to have comorbid medical problems, functional decline, and cognitive impairment, IPV may be particularly devastating. However, despite calls by experts for increased research on IPV in middle-aged and older women,^7,8,9,10,11^ few studies have examined IPV in this population, leading the US Preventive Services Task Force to find insufficient evidence to recommend IPV screening beyond childbearing years.^12^

However, experience of IPV may persist into or begin in older adulthood. A study surveying 370 women revealed that 26.5% of those aged 65 years and older had experienced lifetime IPV and 3.5% had experienced IPV in the past 5 years.^8^ Among 91 749 women aged 50 to 79 years surveyed in the Women’s Health Initiative, 5% reported new experiences of IPV in the previous 3 years.^13^ These studies and others suggest that women experiencing IPV in later life may have experienced abuse throughout their lifetime^9,11^ and continue to experience repeated abuse in older age.^10^ A telephone survey study of a nationally representative sample of women veterans receiving primary care in VHA found declining rates of past-year IPV with age, but substantial proportions of women reported IPV in middle and older age.^14^ These prior studies of older women have been limited to assessing IPV prevalence via survey methodology, which may result in greater disclosure than through clinical screening.

Recent universal IPV screening policies in the VHA present a new opportunity to evaluate IPV screening and outcomes among older women. Women veterans currently account for 6.5% of the VHA patient population and are the fastest-growing sector of VHA patients.^15^ Despite the US Preventive Services Task Force IPV screening recommendations that limit screening to women of reproductive age,^16^ the VHA began widespread implementation of universal IPV screening for women patients of all ages in 2014, using the Extended–Hurt Insult Threaten Scream (E-HITS) tool.^17^

Given the potentially substantial proportion of older women who may continue to experience IPV in later life or for the first time in older age, the lack of data on whether health consequences associated with IPV in younger women are similar or more intense among older women, and the anticipated increase in the older adult population in the US,^18^ this study aimed to determine the proportion of women screening positive for IPV among a universally screened cohort of women VHA patients aged 45 years and older and to examine the association of screening positive for past-year IPV with subsequent diagnoses and health care service utilization.

## Methods

Approval for this study was granted by the Corporal Michael J. Crescenz VA Medical Center institutional review board, which waived the requirement for informed consent because this study used deidentified medical record data. This study follows the Strengthening the Reporting of Observational Studies in Epidemiology (STROBE) reporting guideline.^19^

### Study Sample

The study sample included all women patients with documentation of a completed E-HITS screen in their VHA medical records from April 2014 to April 2016. Annual screens were triggered via electronic reminders in the Veterans Affairs (VA) medical record and were mostly completed in primary care and women’s health clinics by nurses or physicians. Most women in the sample (3565 of 4481 [79.6%]) had only 1 completed screening during the study period. For women with multiple documented IPV screenings, the index screening was defined as the first positive result or, if no positive results, the first screening. This included 4481 women VHA patients aged 45 years and older screened at 1 of 13 VA facilities in 11 US states.

### Measures

Data for this study were obtained from the VA Corporate Data Warehouse, a repository of VHA electronic health records aggregated from all VHA facilities nationwide. We extracted clinical diagnoses and VHA health service utilization during the 20-month period following the index screening date, which was the longest amount of time with complete data available for the full cohort.

#### Baseline Characteristics

Sociodemographic factors extracted included age, race/ethnicity, and marital status. Veteran status (given that some VHA facilities provide care to nonveteran spouses or dependents of eligible veterans) and data on combat exposure and history of military sexual trauma (defined as sexual assault and/or repeated, threatening sexual harassment during military service^20^) were also extracted for use in sensitivity analyses. Age was classified as middle-aged (aged 45-59 years) and older (aged ≥60 years).

#### IPV

The E-HITS tool^17,21^ was designed to be administered in the outpatient setting and is composed of the following 5 categories, with responses from 1, indicating never, to 5, indicating frequently: “How often in the past year has a current of former partner: (1) physically hurt you, (2) insulted or talked down to you, (3) threatened you with harm, (4) screamed or cursed at you, or (5) forced you to have sexual activities?” Total scores range from 5 to 25, with a score of 7 or greater indicating a positive IPV screening based on a prior study validating E-HITS in women VHA patients.^22^ We further classified E-HITS responses into 4 previously defined mutually exclusive categories,^23,24^ as follows: none (no IPV), psychological abuse only, physical abuse without forced sex, and any sexual abuse.

#### Health Conditions and Utilization

Mental and physical health diagnoses were determined by *International Classification of Diseases, Ninth Edition *(*ICD*-*9*) and *ICD*-*10* diagnostic codes assigned to at least 1 inpatient or at least 2 outpatient encounters (separated by ≥30 days) in VA medical records during the 20 months following the index IPV screening for each patient. We used modified Elixhauser^25,26^ and Agency for Healthcare Research and Quality Clinical Classification Software^27^ groupings to classify mental and physical health conditions. We included 5 mental health diagnostic groupings, as follows: anxiety, PTSD, depression, substance use disorder (combined alcohol and drug), and suicidal ideation and/or suicidal or self-harm behaviors.^23^ We included 9 physical health diagnostic groupings that have either been previously shown to be associated with IPV in younger women or were hypothesized to be associated with IPV, as follows: chronic pain, hypertension, nausea and/or vomiting, other gastrointestinal tract disorders, noninfectious genitourinary disorders, urinary tract infections, headache, injuries and/or burns, and skin ulcers and/or infections. Health service utilization during the 20-month postscreening period was based on inpatient and outpatient encounters generated with each service and categorized into psychosocial visits (ie, mental health, social work, drug or alcohol treatment, and homeless services), primary care visits, emergency department visits, specialty outpatient visits (eg, cardiology, rheumatology), and any inpatient admission.

### Statistical Analysis

Baseline characteristics, diagnoses, and utilization were compared across IPV screening status and age categories. Inpatient utilization was analyzed as categorical (ie, any or none) because of overall low rates of inpatient admissions. Separate logistic regression models for each age category were used to assess the associations between IPV screening status and outcomes of diagnosis or any inpatient admission. Because the outpatient utilization counts were overdispersed, negative binomial regression with a log-link was used to model the rate of encounters for each health service type by IPV status during the 20-month follow-up period. We first computed models that only included the primary exposure of interest (ie, positive IPV screening vs negative IPV screening). We then computed models adjusted for age and race/ethnicity (diagnosis models) and then age, race/ethnicity, and marital status (utilization models). In all models, robust standard errors using the sandwich estimator were used to account for potential clustering by VA facility. In sensitivity analyses, models restricted to veteran patients and adjusted for history of military sexual trauma and combat exposure were computed.^28,29,30^ In these models, estimates for the primary exposure of interest were found to be very similar to the main models, so are not reported. In exploratory analyses, psychological-only IPV and IPV involving physical or sexual abuse were examined separately; because of small numbers, these analyses were considered hypothesis-generating (eTable 1 and eTable 2 in the Supplement).

All analyses were performed using R statistical computing software version 3.6.2 (R Project for Statistical Computing). Statistical significance was set at *P* < .05, and all tests were 2-tailed.

## Results

Of 4481 women VHA patients (1955 [43.6%] black), 2937 (65.5%) were middle-aged (ie, aged 45-59 years) and 1544 (34.5%) were older (ie, aged ≥60 years). Baseline characteristics are presented in Table 1. A higher proportion of middle-aged women had black, other, or unknown race compared with older women (1693 [57.6%] vs 698 [45.2%]), but both groups were racially diverse. Most of those screened in both age groups were veterans (middle-aged, 2742 [93.4%]; older, 1352 [87.6%]). A total of 255 middle-aged women (8.7%) and 79 older women (5.1%) screened positive for past-year IPV. Most of those who screened positive for IPV reported psychological abuse only (middle-aged, 189 [74.1%]; older, 63 [79.7%]). However, 66 middle-aged women (25.9%) and 16 older women (20.3%) experienced physical abuse or forced sex.

**Table 1.** Characteristics of 4481 Women Veterans Screened for Intimate Partner Violence

Characteristic	No. (%) by age group			
	45-59 y (n = 2937)		≥60 y (n = 1544)	
	Screened IPV negative (n = 2682)	Screened IPV positive (n = 255)	Screened IPV negative (n = 1465)	Screened IPV positive (n = 79)
Age at screen, mean (SD), y	52 (4)	51 (4)	67 (8)	64 (5)
Race/ethnicity				
Black	1294 (48.2)	105 (41.2)	523 (35.7)	33 (41.8)
Non-Hispanic white	1125 (41.9)	119 (46.7)	806 (55.0)	40 (50.6)
Other	144 (5.4)	22 (8.6)	75 (5.1)	2 (2.5)
Unknown	119 (4.4)	9 (3.5)	61 (4.2)	4 (5.1)
Marital status				
Divorced, separated, or widowed	1141 (42.5)	104 (40.8)	705 (48.1)	30 (38.0)
Married	980 (36.5)	119 (46.7)	542 (37.0)	45 (57.0)
Never married	535 (19.9)	31 (12.2)	208 (14.2)	3 (3.8)
Unknown	26 (1.0)	1 (0.4)	10 (0.7)	1 (1.3)
Veteran status				
Veteran	2516 (93.8)	226 (88.6)	1295 (88.4)	57 (72.2)
Nonveteran	166 (6.2)	29 (11.4)	170 (11.6)	22 (27.8)
Combat service^a^	180 (7.2)	23 (10.2)	35 (2.7)	0
History of military sexual trauma^a^	694 (27.6)	100 (44.2)	275 (21.2)	23 (40.4)
IPV type				
None	2682 (91.3)		1465 (94.9)	
Psychological abuse only	189 (6.4)		63 (4.1)	
Physical abuse without forced sex	41 (1.4)		12 (0.8)	
Any sexual abuse	25 (0.9)		4 (0.3)	

Abbreviation: IPV, intimate partner violence.

^a^ Combat service and history of military sexual trauma includes veterans only.

Those screening positive for IPV in both groups were more likely than those screening negative to be diagnosed with each category of mental health condition (Table 2). For example, 446 older women (30.4%) who screened negative for IPV had a depression diagnosis in the subsequent 20 months compared with 47 older women (59.5%) who screened positive (*P* < .001). The proportions of women in both age groups diagnosed with physical health conditions were more similar by IPV status. Compared with middle-aged women who screened negative for IPV, those who screened positive for IPV were more likely to have any inpatient admission in the subsequent 20-month period (304 [11.3%] vs 51 [20.0%]; *P* < .001) and to have a higher median (interquartile range) number of primary care visits (9 [5-15] visits vs 11 [6-17.5] visits; *P* = .002), psychosocial visits (3 [0-13] visits vs 11 [2-36] visits; *P* < .001), and emergency department visits (0 [0-2] visits vs 1 [0-2] visits; *P* < .001) (Table 2). Among older women, those who screened positive for IPV had a greater median (interquartile range) number of psychosocial visits than those who screened negative for IPV (1 [0-6] visits vs 4 [1-14] visits; *P* < .001).

**Table 2.** Medical Diagnoses and Healthcare Utilization in the 20 Months After Screening by IPV Status

Outcome	Women aged 45-59 y, No. (%)		*P* value^a^	Women aged ≥60 y, No. (%)		*P* value^a^
	Screened IPV negative (n = 2682)	Screened IPV positive (n = 255)		Screened IPV negative (n = 1465)	Screened IPV positive (n = 79)	
Medical condition						
Anxiety	554 (20.7)	90 (35.3)	<.001	199 (13.6)	18 (22.8)	.03
Depression	1174 (43.8)	164 (64.3)	<.001	446 (30.4)	47 (59.5)	<.001
PTSD	671 (25)	112 (43.9)	<.001	163 (11.1)	18 (22.8)	.003
Substance use disorder	240 (8.9)	51 (20)	<.001	71 (4.8)	9 (11.4)	.02
Suicidal ideation and/or behavior	43 (1.6)	15 (5.9)	<.001	13 (0.9)	1 (1.3)	>.99
Chronic pain	570 (21.3)	49 (19.2)	.50	227 (15.5)	13 (16.5)	.94
Hypertension	903 (33.7)	88 (34.5)	.84	730 (49.8)	38 (48.1)	.85
Nausea and vomiting	65 (2.4)	17 (6.7)	<.001	39 (2.7)	1 (1.3)	.69
Other GI tract disorder	354 (13.2)	47 (18.4)	.03	222 (15.2)	11 (13.9)	.89
Noninfectious GU disorder	445 (16.6)	59 (23.1)	.01	229 (15.6)	13 (16.5)	.97
Urinary tract infection	34 (1.3)	3 (1.2)	>.99	27 (1.8)	2 (2.5)	.99
Headache	538 (20.1)	61 (23.9)	.17	123 (8.4)	14 (17.7)	.008
Injuries and burns	247 (9.2)	31 (12.2)	.15	95 (6.5)	10 (12.7)	.06
Skin ulcers or infection	156 (5.8)	18 (7.1)	.51	103 (7.0)	12 (15.2)	.01
Health care visits, median (IQR)						
Psychosocial	3 (0-13)	11 (2-36)	<.001	1 (0-6)	4 (1-14)	<.001
Primary care	9 (5-15)	11 (6-17.5)	.002	8 (4-15)	9 (5.5-17)	.07
Specialty outpatient	2 (0-6)	2 (0-7)	.18	1 (0-6)	2 (0-6)	.20
Emergency department	0 (0-2)	1 (0-2)	<.001	0 (0-1)	0 (0-1)	.68
Inpatient admissions	304 (11.3)	51 (20.0)	<.001	195 (13.3)	11 (13.9)	>.99

Abbreviations: GI, gastrointestinal; GU, genitourinary; IPV, intimate partner violence; IQR, interquartile range; PTSD, posttraumatic stress disorder.

^a^ *P* values from χ^2^ tests when comparing proportions or from Wilcoxon rank sum nonparametric tests when comparing medians.

In adjusted logistic regression models for the middle-aged group, screening positive for IPV was significantly associated with anxiety (adjusted odds ratio [aOR], 2.00; 95% CI, 1.50-2.70; *P* < .001), depression (aOR, 2.30; 95% CI, 1.80-3.00; *P* < .001), PTSD (aOR, 2.30; 95% CI, 1.80-3.00; *P* < .001), suicidal ideation and/or behavior (aOR, 3.80; 95% CI, 2.10-6.90; *P* < .001), and substance use disorder (aOR, 2.50; 1.80-3.50; *P* < .001) (Figure). Significant associations were also seen for nausea and/or vomiting (aOR, 2.90; 95% CI, 1.70-5.00; *P* < .001), other gastrointestinal tract disorders (aOR, 1.50; 95% CI, 1.10-2.10; *P* = .02), and noninfectious genitourinary disorders (aOR, 1.50; 95% CI, 1.10-2.00; *P* = .02). No significant associations were seen between screening positive for IPV and other health conditions studied.

**Figure.**
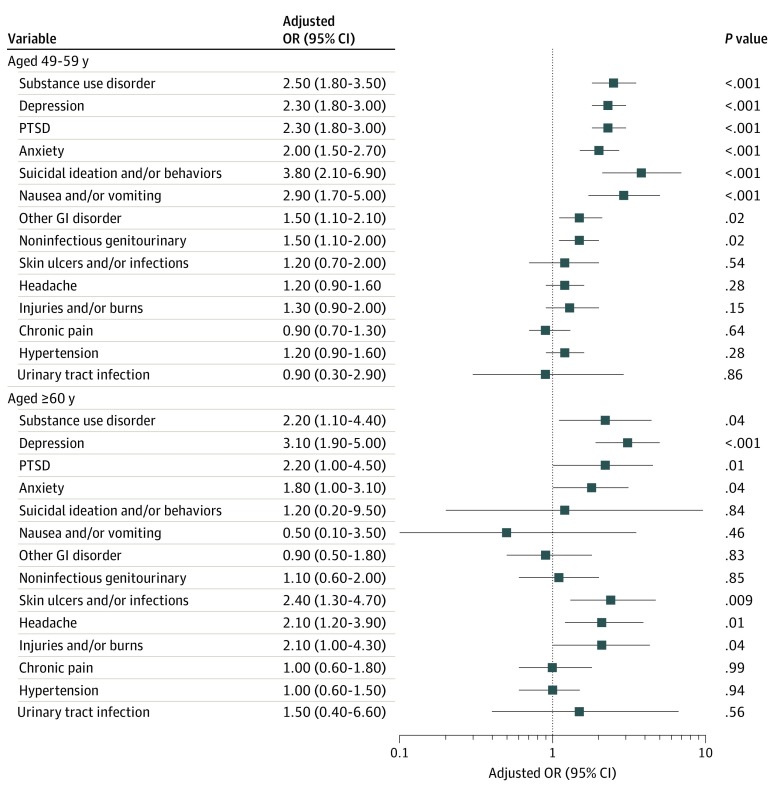
Association of Intimate Partner Violence With Mental and Physical Health Conditions in Women Aged 45 to 59 Years and Those Aged 60 Years and Older GI indicates gastrointestinal; OR, odds ratio; and PTSD, posttraumatic stress disorder.

For older women, significant associations remained for screening positive for IPV with all mental health conditions except suicidal ideation/behavior, which had a small count and large confidence interval (anxiety: aOR, 1.80; 95% CI, 1.00-3.10; *P* = .04; depression: aOR, 3.10; 95% CI, 1.90-5.00; *P* < .001; PTSD: aOR, 2.20; 95% CI, 1.00-4.50; *P* = .01; substance use disorder: aOR, 2.20; 95% CI, 1.10-4.40; *P* = .04; suicidal ideation and/or behavior: aOR, 1.20; 95% CI, 0.20-9.50; *P* = .84). Older women had a different pattern of associations between screening positive for IPV and physical health conditions. Screening positive for IPV was associated with increased odds of headache (aOR, 2.10; 95% CI, 1.20-3.90; *P* = .01), injuries and burns (aOR, 2.10; 95% CI, 1.00-4.30; *P* = .04), and skin ulcer or infection (aOR, 2.40; 95% CI, 1.30-4.70; *P* = .009) (Figure).

Screening positive for IPV was associated with an increased rate of psychosocial visits for both middle-aged and older women (Table 3). Compared with middle-aged women who screened negative for IPV, those who screened positive had more than double the rate of psychosocial visits in the subsequent 20 months (adjusted rate ratio [aRR], 2.40; 95% CI 2.00-2.90; *P* < .001), and older women who screened positive had nearly double the rate of those who screened negative (aRR, 1.90; 95% CI, 1.30-2.70; *P* < .001). Middle-aged women who screened positive for IPV also had increased rates of primary care visits (aRR, 1.20; 95% CI, 1.10-1.30; *P* = .003) and emergency department visits (aRR, 1.50; 95% CI, 1.20-1.80; *P* < .001) as well as higher odds of having any inpatient admission (aOR, 2.10; 95% CI, 1.50-2.90; *P* < .001) compared with those who screened negative. These associations were not seen for the older women (Table 3).

**Table 3.** Association of Screening Positive for Intimate Partner Violence With Health Service Utilization in the Subsequent 20 Months Among Middle-Aged and Older Women

Health service	RR (95% CI)		*P* value
	Unadjusted	Adjusted^a^	
**Women aged 45-59 y**			
Primary care visits	1.20 (1.10-1.30)	1.20 (1.10-1.30)	.003
Psychosocial visits^b^	2.30 (1.90-2.80)	2.40 (2.00-2.90)	<.001
Specialty outpatient visits^c^	1.00 (0.80-1.20)	1.00 (0.80-1.20)	.93
ED visits	1.50 (1.20-1.80)	1.50 (1.20-1.80)	<.001
Any inpatient admission, OR (95% CI)	2.00 (1.40-2.70)	2.10 (1.50-2.90)	<.001
**Women aged ≥60 y**			
Primary care visits	1.10 (0.90-1.30)	1.10 (1.00-1.30)	.15
Psychosocial visits^b^	1.70 (1.20-2.30)	1.90 (1.30-2.70)	<.001
Specialty outpatient visits^c^	1.10 (0.70-1.70)	1.10 (0.80-1.60)	.69
ED visits	1.10 (0.70-1.70)	1.10 (0.70-1.60)	.82
Any inpatient admission, OR (95% CI)	1.10 (0.60-2.00)	1.20 (0.60-2.30)	.56

Abbreviations: ED, emergency department; OR, odds ratio; RR, rate ratio.

^a^ Adjusted for age, race/ethnicity, and marital status.

^b^ Psychosocial visits include mental health, social work, drug or alcohol treatment, and homeless services.

^c^ Specialty outpatient visits included medical subspecialties (eg, cardiology, rheumatology).

## Discussion

In this study evaluating clinical past-year IPV screening responses among more than 4000 women older than childbearing age who were seen in VHA outpatient settings, we identified a substantial proportion of documented positive IPV screening results and associations with subsequent mental health conditions and psychosocial health service utilization. Nearly 9% of middle-aged women and more than 5% of older women screened positive for IPV in this study compared with 10% of women VHA patients younger than 45 years who were screened at the same sites during the same period.^31^ These findings point to potential benefits of extending routine clinical IPV screening beyond reproductive age.

Our findings build on a growing body of knowledge on the association of interpersonal violence with health in women older than reproductive age. Prior studies have examined the consequences of lifetime IPV exposure on health for women in midlife and older and found associations with depression and anxiety,^9,10,32,33,34^ gastrointestinal tract disorders,^10,35^ menopausal disorders,^36^ cardiovascular risk factors,^10^ chronic pain,^10,35^ and functional decline.^37^ Our study adds to this work by demonstrating that more proximal IPV experience (ie, in the past year) in midlife and older age has near-term associations with both mental and physical health outcomes. To our knowledge, this study is also the first to assess the association between IPV and health via routine screening of women who are postreproductive age in clinical practice.

Similar to prior studies among younger women,^2,3^ our study found a strong association between experiencing IPV and mental health conditions. In particular, most women who screened positive for IPV in both age groups received a depression diagnosis in the subsequent 20-month period. Older women had more than 3 times the odds of having a depression diagnosis if they screened positive for IPV. Middle-aged women screening positive had more than twice the odds of having a diagnosis of depression, anxiety, PTSD, or substance use disorder, and nearly 4 times the odds of having suicidal behaviors or self-harm. These associations suggest the critical importance of addressing IPV when treating middle-aged and older women for mental health conditions; failing to assess for IPV may result in missing a key contributor.

Numerous studies have demonstrated increased utilization of health services for younger women who have experienced IPV^4,6,38^; our study demonstrates similar findings for middle-aged and older women, with a particularly pronounced difference in the rate of visits related to psychosocial care. These visits present important opportunities for health care providers to address IPV-related concerns in the health care setting. Literature on younger women has described many benefits of addressing IPV in health care settings^39^; for middle-aged and older women, approaches and services should be tailored to the specific needs, generational culture, and health-related issues connected with their experience of IPV. Fewer associations with health care utilization were seen in older women compared with middle-aged women. This may be because older VHA patients have higher non-VHA medical service use because of Medicare eligibility (ie, dual use)^40,41^ or because those experiencing IPV have increased access barriers or avoidance of care. Future studies that link VA and non-VA data sources may be able to further explore this association in the Medicare-eligible VHA population.

This study evaluated IPV experience in middle-aged and older women separately, given that each stage of aging brings both physiologic and increasingly frequent pathologic changes that may affect the risk of experiencing IPV as well as the consequences of this experience. For example, we found that for older women, but not middle-aged women, screening positive for IPV was associated with injuries and skin ulcers, which may result from increasing frailty, skin thinning, sarcopenia, or osteoporosis as women age. The consequences of experiencing IPV may also be different for women older than 60 years, given that this is the age at which most states define abuse as elder abuse and require mandatory reporting by health care providers. Changes in cognitive function, leading to mild cognitive impairment and dementia, may also affect both susceptibility to experiencing violence as well as the likelihood of using violence in intimate relationships owing to caregiver dynamics and stress. While we did not have adequate numbers in this study to assess associations between dementia and IPV—and dementia can present challenges to self-report screening—studies on elder abuse have found dementia to be a significant risk factor.^42,43,44^ Further studies could elucidate the contribution of cognitive impairment to IPV risk, both as couples age and as individuals seek new relationships in later life.

Most of the IPV reported in this study was classified as psychological abuse only. This is notable given popularized beliefs that IPV refers to physical and sexual abuse. However, our findings demonstrate that nonphysical forms of abuse were significantly associated with adverse mental health in middle-aged and older women. This highlights the importance of communicating to older women, who may have grown up when gender roles and relationship dynamics were measured against different norms, that psychological violence can have devastating health consequences. These findings are consistent with work in elder abuse that has similarly found that psychological mistreatment of older adults has severe negative health consequences.^45^

### Strengths and Limitations

This study has strengths, such as looking at IPV identified via real-world routine clinical screening rather than a study evaluation tool, including a relatively large number of middle-aged and older women, and collecting data on several health-related outcomes. This study also has limitations, such as the temporal imprecision of the exposure and outcomes and the possibility of reverse causality in this observational study. To control for this as much as possible, we only assessed diagnoses and health care utilization that occurred on or following the screening date, even though IPV could have occurred any time in the year before the screening. However, because this study relied on medical record data, we could only ascertain the date of diagnosis for health conditions studied, not the date of onset. Furthermore, this study did not include diagnoses or care received outside of the VHA.

The population studied, ie, women VHA patients, may not reflect the more general US population, and IPV prevalence may be higher among women veterans than nonveterans.^46^ Additionally, although universally applied, IPV screening may not have included all eligible patients, and we were not able to ascertain potential differences between those who did and did not complete IPV screening. Men were not included in this study but may also experience IPV^47^ and should be included in future studies. In addition, while we studied older female patients, the mean age of the older group was still relatively young (67 years) and may not represent the experience of women in their 70s, 80s, and older. This study was an important step toward understanding the utility and importance of IPV screening in older age groups, laying the foundation for future studies that expand the study population.

## Conclusions

In this study, routine screening for IPV in clinical practice detected a substantial number of positive results among middle-aged and older women. Screening positive for IPV was associated with both mental and physical health conditions in these age groups as well as significantly higher downstream health service utilization. Screening for IPV after childbearing age presents an opportunity to identify a high-risk population that may benefit from interventions and services based in health care settings to improve outcomes.

## Notes

Supplement. **eTable 1.** Association of Screening Positive for Psychological-Only IPV or IPV Involving Physical or Sexual Abuse With Health Conditions Diagnosed in the Subsequent 20 Months for Middle-Aged and Older Women **eTable 2.** Association of Screening Positive for Psychological-Only IPV or IPV Involving Physical or Sexual Abuse With Health Service Utilization in the Subsequent 20 Months for Middle-Aged and Older Women
