# Supplementary material for: Association of Health Conditions and Health Service Utilization With Intimate Partner Violence Identified via Routine Screening Among Middle-Aged and Older Women
Source: JAMA Netw Open. 2020 Apr 21;3(4):e203138. doi: 10.1001/jamanetworkopen.2020.3138 (PMC7175082; doi:10.1001/jamanetworkopen.2020.3138)
Supplement: Supplement. — eTable 1. Association of Screening Positive for Psychological-Only IPV or IPV Involving Physical or Sexual Abuse With Health Conditions Diagnosed in the Subsequent 20 Months for Middle-Aged and Older Women eTable 2. Association of Screening Positive for Psychological-Only IPV or IPV Involving Physical or Sexual Abuse With Health Service Utilization in the Subsequent 20 Months for Middle-Aged and Older Women [file jamanetwopen-3-e203138-s001.pdf]

Supplementary Online Content

Makaroun LK, Brignone E, Rosland A-M, Dichter ME. Association of health conditions and health service utilization with intimate partner violence identified via routine screening among middle-aged and older women. *JAMA Netw Open*. 2020;3(4): e203138. doi: 10.1001/jamanetworkopen.2020.3138

eTable 1. Association of Screening Positive for Psychological-Only IPV or IPV Involving Physical or Sexual Abuse With Health Conditions Diagnosed in the Subsequent 20 Months for Middle-Aged and Older Women
eTable 2. Association of Screening Positive for Psychological-Only IPV or IPV Involving Physical or Sexual Abuse With Health Service Utilization in the Subsequent 20 Months for Middle-Aged and Older Women

This supplementary material has been provided by the authors to give readers additional information about their work.

© 2020 Makaroun LK et al. *JAMA Network Open.*

eTable 1. Association of Screening Positive for Psychological-Only IPV or IPV Involving Physical or Sexual Abuse With Health Conditions Diagnosed in the Subsequent 20 Months for Middle-Aged and Older Women

**Age 45-59 y**	**IPVPsy+ vs. IPV-**		**IPVPhSx+ vs. IPV-**	
**Health Condition**	**Adjusted[a] OR (95% CI)**	**p-value**	**Adjusted OR (95% CI)**	**p-value**
Anxiety	1.6 (1.2 – 2.2)	0.005*	3.8 (2.3 – 6.3)	<0.001*
Depression	2.0 (1.5 – 2.7)	<0.001*	3.8 (2.2 – 6.6)	<0.001*
PTSD	2.2 (1.6 – 3.0)	<0.001*	2.5 (1.5 – 4.1)	<0.001*
Suicidal ideation/behavior	3.1 (1.5 – 6.4)	0.002*	6.1 (2.5 – 14.7)	<0.001*
Substance abuse	1.8 (1.2 – 2.7)	0.01*	5.5 (3.2 – 9.3)	<0.001*
Chronic pain	0.9 (0.6 – 1.3)	0.44	1.1 (0.6 – 2.0)	0.69
Headache	1.1 (0.8 – 1.6)	0.50	1.3 (0.8 – 2.3)	0.30
Hypertension	1.1 (0.8 – 1.5)	0.73	1.5 (0.9 – 2.5)	0.10
Nausea/vomiting	2.7 (1.5 – 5.2)	0.002*	3.4 (1.3 – 8.6)	0.01*
Other GI disorder	1.1 (0.8 – 1.7)	0.55	2.7 (1.6 – 4.7)	<0.001*
Non-infectious GU disorder	1.3 (0.9 – 1.9)	0.10	1.8 (1.0 – 3.1)	0.051
Urinary tract infection	1.3 (0.4 – 4.1)	0.71	n/a	n/a
Injuries and burns	1.1 (0.7 – 1.8)	0.63	2.0 (1.0 – 3.9)	0.04*
Skin ulcer or infection	1.2 (0.7 – 2.2)	0.47	1.0 (0.4 – 2.8)	0.99
**Age ≥ 60 y**	**IPVPsy+ vs. IPV-**		**IPVPhSx+ vs. IPV-**	
**Health Condition**	**Adjusted OR (95% CI)**	**p-value**	**Adjusted OR (95% CI)**	**p-value**
Anxiety	1.8 (1.0 – 3.4)	0.054	1.6 (0.5 – 5.4)	0.48
Depression	3.4 (2.0 – 5.7)	<0.001*	2.3 (0.7 – 7.2)	0.15
PTSD	2.4 (1.3 – 4.3)	0.004*	1.1 (0.2 – 5.2)	0.93
Suicidal ideation/behavior	1.5 (0.2 – 11.6)	0.71	n/a[b]	n/a
Substance abuse	2.5 (1.1 – 5.5)	0.02*	1.0 (0.1 – 7.4)	0.98
Chronic pain	1.1 (0.6 – 2.1)	0.81	0.7 (0.2 – 2.9)	0.62
Headache	1.9 (0.9 – 3.8)	0.09	3.5 (1.1 – 10.9)	0.03*
Hypertension	1.1 (0.7 – 1.8)	0.64	0.6 (0.2 – 1.6)	0.30
Nausea/vomiting	0.6 (0.08 – 4.4)	0.61	n/a	n/a
Other GI disorder	1.0 (0.5 – 2.0)	0.88	0.9 (0.2 – 3.9)	0.84
Non-infectious GU disorder	1.0 (0.5 – 2.0)	0.99	1.3 (0.4 – 4.6)	0.68
Urinary tract infection	1.0 (0.1 – 7.3)	0.97	3.8 (0.5 – 31.0)	0.21
Injuries and burns	2.1 (0.9 – 4.6)	0.08	2.3 (0.5 – 10.5)	0.28
Skin ulcer or infection	2.5 (1.2 – 5.1)	0.01*	2.1 (0.5 – 9.9)	0.35

© 2020 Makaroun LK et al. *JAMA Network Open.*

a. All models presented adjusted for age and race/ethnicity
b. n/a: not applicable as cell size = 0
* Statistically significant at the α <0.05 level.
IPVPsy = psychological-only IPV; IPVPhSx = IPV involving physical or sexual abuse; OR = odds ratio; PTSD = Post-traumatic stress disorder; STI = sexually transmitted infection; GI = gastrointestinal; GU = genitourinary

© 2020 Makaroun LK et al. *JAMA Network Open.*

eTable 2. Association of Screening Positive for Psychological-Only IPV or IPV Involving Physical or Sexual Abuse With Health Service Utilization in the Subsequent 20 Months for Middle-Aged and Older Women

**Age 45-59 y**	**IPVPsy+ vs. IPV-**		**IPVPhSx+ vs. IPV-**	
**Health service**	**Adjusted[a] RR (95% CI)**	**p-value**	**Adjusted RR (95% CI)**	**p-value**
Primary care visits	1.1 (1.0 – 1.2)	0.15	1.4 (1.2 – 1.7)	0.001*
Psychosocial visits	2.0 (1.6 – 2.5)	<0.001*	3.5 (2.5 – 4.8)	<0.001*
Specialty outpatient visits	0.9 (0.8 – 1.2)	0.63	1.1 (0.8 – 1.5)	0.44
ED visits	1.3 (1.0 – 1.6)	0.12	2.1 (1.6 – 2.9)	<0.001*
	**Adjusted OR (95% CI)**	**p-value**	**Adjusted OR (95% CI)**	**p-value**
Any inpatient admission	1.9 (1.3 – 2.9)	0.001*	2.6 (1.4 – 4.7)	0.002*
**Age ≥ 60 y**	**IPVPsy+ vs. IPV-**		**IPVPhSx+ vs. IPV-**	
**Health Service**	**Adjusted RR (95% CI)**	**p-value**	**Adjusted RR (95% CI)**	**p-value**
Primary care visits	1.1 (0.9 – 1.3)	0.25	1.2 (0.8 – 2.0)	0.35
Psychosocial visits	1.9 (1.3 – 2.9)	0.001*	1.7 (0.7 – 4.1)	0.23
Specialty outpatient visits	1.2 (0.8 – 1.8)	0.40	0.6 (0.3 – 1.4)	0.27
ED visits	1.0 (0.6 – 1.6)	1.0	1.2 (0.6 – 2.6)	0.56
	**Adjusted OR (95% CI)**	**p-value**	**Adjusted OR (95% CI)**	**p-value**
Any inpatient admission	1.4 (0.7 – 2.9)	0.31	0.5 (0.06 – 3.6)	0.47

a. All models presented adjusted for age, race/ethnicity and marital status
* Statistically significant at the α <0.05 level.
IPVPsy = psychological-only IPV; IPVPhSx = IPV involving physical or sexual abuse; RR = rate ratio; ED = emergency department; OR = odds ratio.
Psychosocial visits include mental health, social work, drug or alcohol treatment and homeless services; Specialty outpatient visits include medical subspecialties (e.g. cardiology, rheumatology).

© 2020 Makaroun LK et al. *JAMA Network Open.*
